# Editorial: Genetics and Molecular Mechanisms of Oral and Esophageal Squamous Cell Carcinoma

**DOI:** 10.3389/fonc.2022.874353

**Published:** 2022-04-06

**Authors:** Bin Qiao, Shuaize Li, Die Wang, Di Wu

**Affiliations:** ^1^ Department of Stomatology, The First Affiliated Hospital of Zhengzhou University, Zhengzhou, China; ^2^ Centre for Cancer Research, Hudson Institute of Medical Research, Melbourne, VIC, Australia; ^3^ Department of Periodontology, School of Dentistry, The University of North Carolina at Chapel Hill, Chapel Hill, NC, United States

**Keywords:** oral cancer, precision medicine, genome sequencing, whole exome sequencing, targeted therapy

Oral squamous cell carcinoma (OSCC) is the most common histopathological type of oral cancer, with typical characteristics of low 5-year survival rate and poor prognosis (1, 2). Importantly, there are many factors affecting its occurrence and progression, in which genome alterations are critical indicators of the proper diagnosis and treatment (3). Carcinogenesis is a multi-step process, which involves the accumulation of genetic and epigenetic changes of oncogenes or tumor suppressor genes (4).Therefore, better understanding of the genetic and molecular disorders of the disease is the key to early diagnosis, appropriate treatment and improving the prognosis of patients.

Nowadays, cancer treatment is developed more towards personalized and targeted treatment. The most widely used treatment are targeted immunotherapy, which have significantly improved the 5-year survival rate of many types of cancer (5, 6). However, for OSCC patients, the only approved targeted therapy is a monoclonal antibody against epidermal growth factor receptor (EGFR), with the trade name ‘cetuximab’ (7). Recently, two immunotherapeutic agents, i.e., pembrolizumab and nivolumab, have been approved for OSCC (8, 9). Nevertheless, patients are widely resistant to the targeted therapy such as cetuximab combined with radiotherapy (10), and only less than 20% of OSCC patients receiving immunotherapy have achieved lasting remission (11). Therefore, it is necessary to implement different treatment schemes for patients based on their different gene mutations. Fortunately, the recent development of high throughput sequencing technologies, including whole genome sequencing and whole exome sequencing, make the detection of gene mutations in tumor tissues more sensitive and comprehensive so that the personalized cancer treatment becomes possible (12). This personalized treatment topic focused on the two genes *EGFR* and *TP53* most commonly mutant in OSCC. In the following, we will demonstrate the rationale and the existing dilemma of targeted therapy of OSCC based on these two genes in the prospect of gene sequencing technology served for precision medicine.

It is reported that *EGFR* is overexpressed in more than 90% of OSCC patients and is involved in tumor cell invasion and metastasis (13). The activation of *EGFR* leads to the phosphorylation and activation of downstream signal transduction mediators and promotes tumor cell proliferation, survival, angiogenesis, invasion and adhesion (14). A variety of strategies to block *EGFR* function have been developed as personalized methods to inhibit tumor growth and metastasis, in which, cetuximab is the only targeted drug approved in OSCC. It has also been widely used and studied in patients with locally advanced OSCC and patients with recurrent and or metastatic OSCC. However, mutations that activate EGFR kinase activity are relatively rare in OSCC (15). In addition, SRC is a nonreceptor tyrosine kinase. It is involved in regulating cell signal transduction downstream of a variety of receptors, including members of EGFR family, and in the regulation of cell proliferation, migration, adhesion and apoptosis (16). SRC kinase activity also enhances EGFR signal transduction (17). Therefore, SRC activity may promote resistance to *EGFR* targeted personalized therapy through independent activation or association with other receptors (17). Therefore, the determination of SRC kinase activity may be the key to predict the possible positive clinical response of targeted therapy.

Another important gene is TP53, which regulates cell cycle and apoptosis induced by DNA damage (18). Studies have shown that TP53 regulates the expression of forkhead box M1 (FOXM1) transcription factor and can directly bind and inactivate Aurora kinase A (AURKA) (19). FOXM1, an important cell cycle mediator, is a transcription factor downstream of EGFR/PI3K/AKT cascade and controls cell survival, apoptosis, migration and angiogenesis (20). In addition, AURKA and AURKB are two cell cycle regulators controlled by FOXM1 (21). AURKA and AURKB both control the structure and function of cytoskeleton and chromosome and contribute to tumor progression, metastasis and diffusion (22). EGFR signaling pathway can improve the translation and transcription efficiency of AURKA and induce the overexpression of AURKA (23). As an important cell cycle regulator, Aurora kinase is a reliable target in a variety of malignant tumors. At present, several Aurora kinase inhibitors have been developed (24, 25). In preclinical evaluation studies, it was found that AURKA and AURKB inhibitors ENMD2076 and AZD1152, as well as pan Aurora agents such as AMG900, can induce growth arrest and apoptosis (26, 27). In a phase I/II study, laser kinase inhibitors were evaluated as a single drug for a variety of solid tumors including OSCC. However, only 3 of 20 OSCC patients receiving AMG900 had partial remission. This low success rate suggests that the overall the personalized intervention needs to be improved at the patient population, and meanwhile those patients who are more sensitive to Aurora kinase inhibitors also need to have further investigator e.g., identify the biomarkers they may share to indicate the efficacy of these compounds, so as to achieve better clinical results from this target therapy.

As a summary, one of the ultimate goals of cancer research is to better understand the disease-related biological process to identify the predictive biomarkers, which runs through the whole process of patient diagnosis, prognosis and treatment. The effect of clinical treatment often depends on the existence of specific cell targets. Despite the complexity of cancer genetics, tumor heterogeneity and drug resistance are still the difficulties of targeted therapy. However, the development of genomics related technologies, including whole genome sequencing and whole exome sequencing, has had a far-reaching impact on the personalized diagnosis and treatment of cancer patients (28).

## Author Contributions

BQ and SL wrote the manuscript. DWa and DWu made the draft revision. All authors contributed to the article and approved the submitted version.

## Conflict of Interest

The authors declare that the research was conducted in the absence of any commercial or financial relationships that could be construed as a potential conflict of interest.

## Publisher’s Note

All claims expressed in this article are solely those of the authors and do not necessarily represent those of their affiliated organizations, or those of the publisher, the editors and the reviewers. Any product that may be evaluated in this article, or claim that may be made by its manufacturer, is not guaranteed or endorsed by the publisher.
